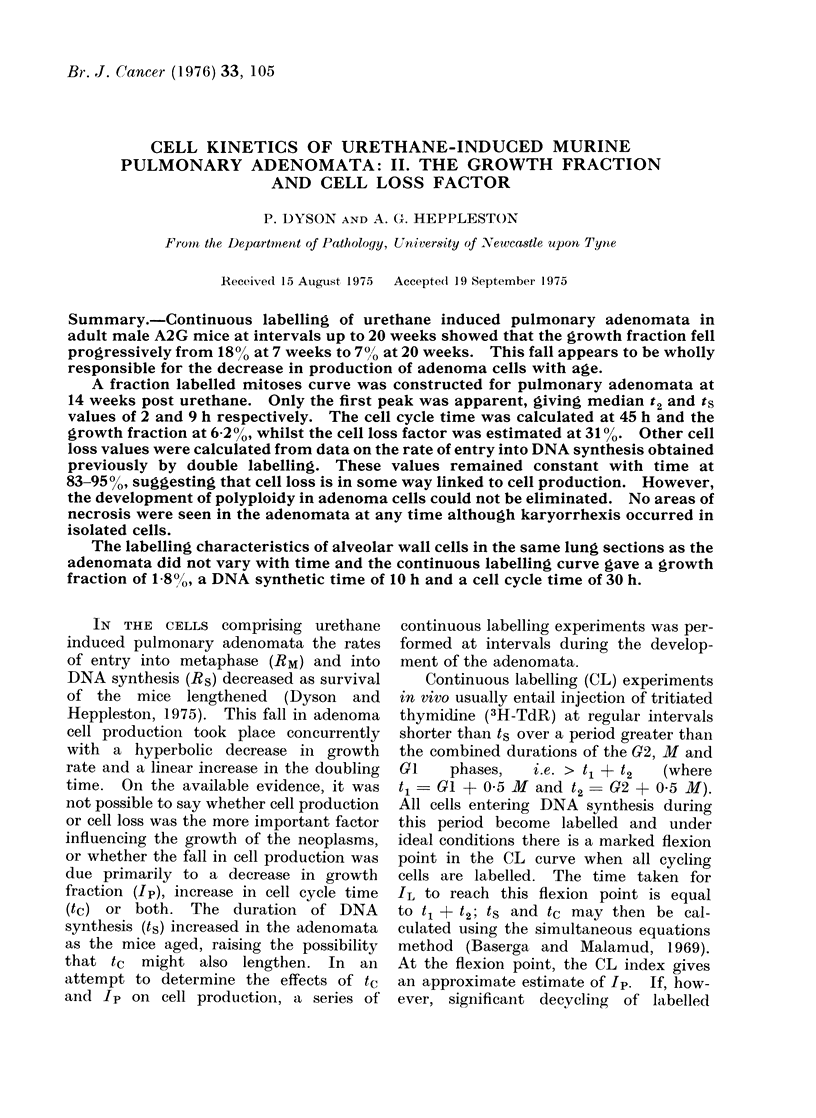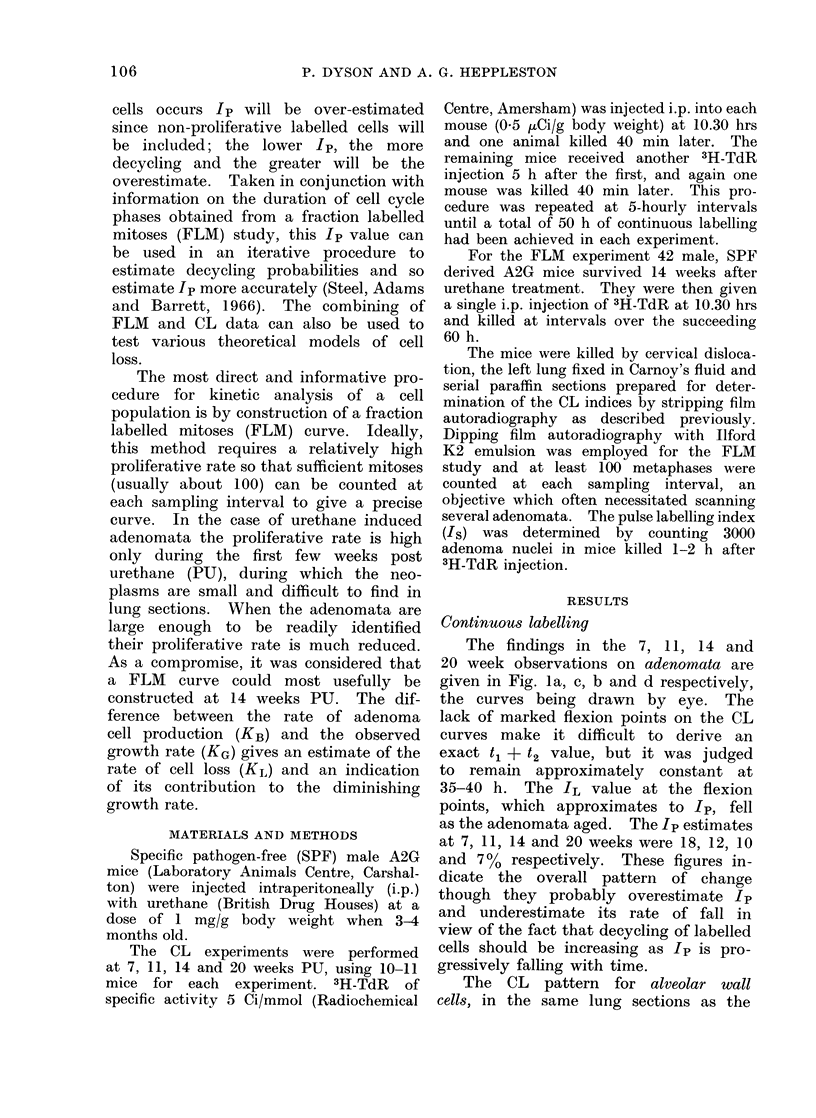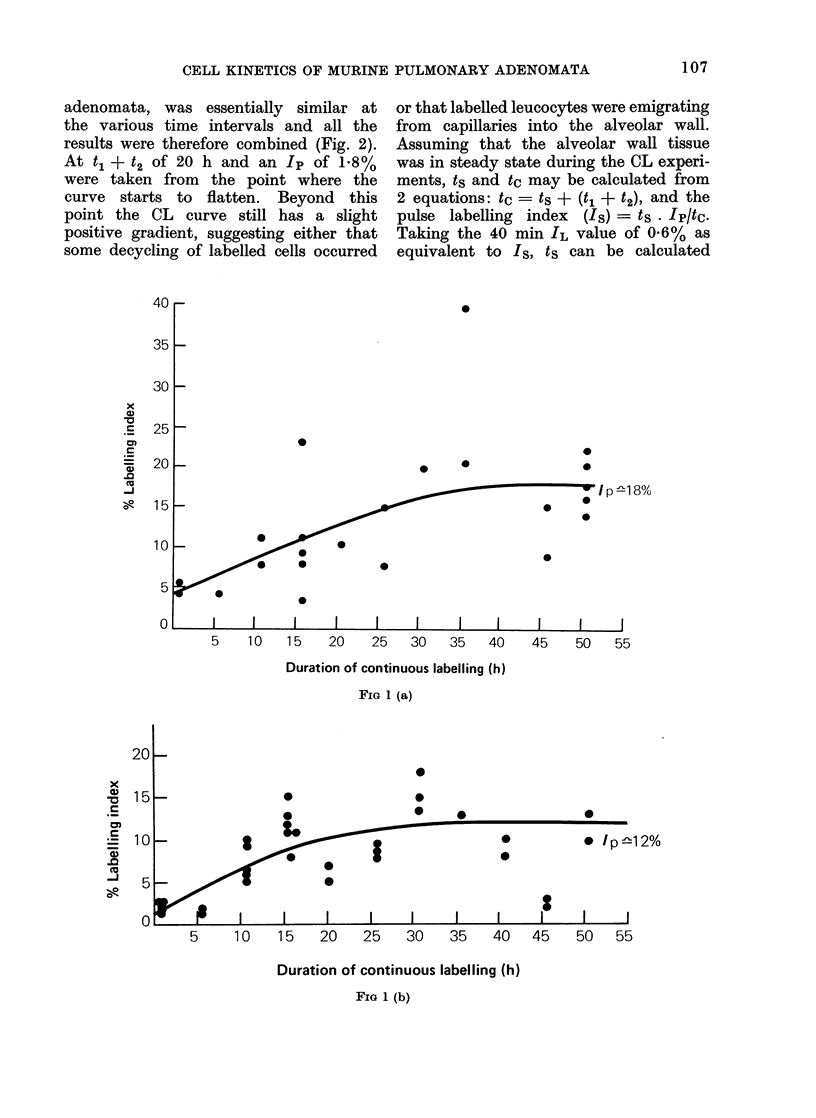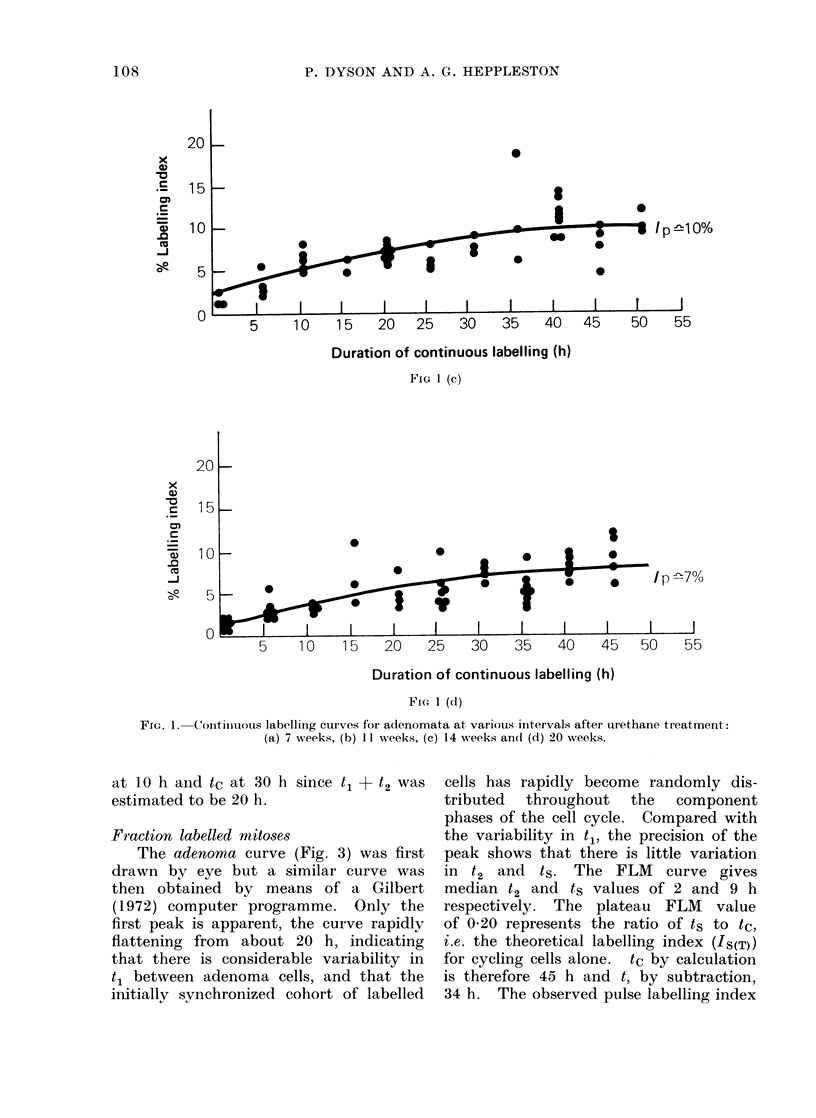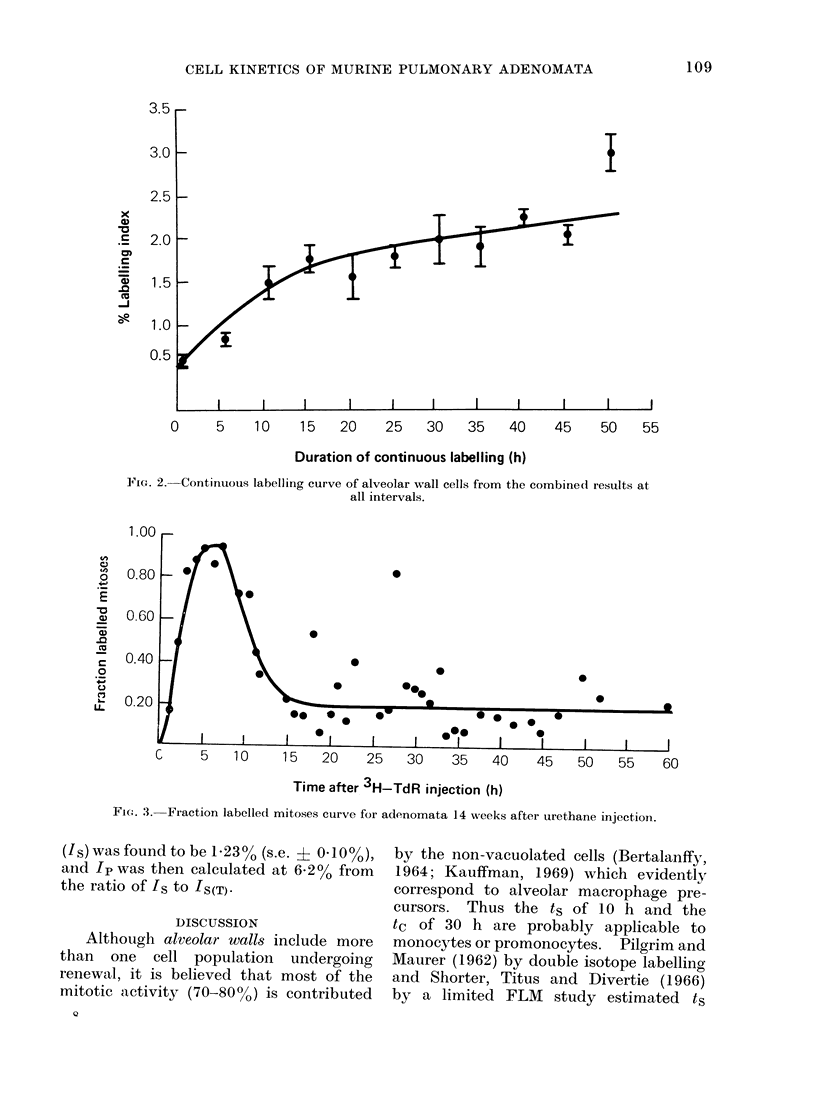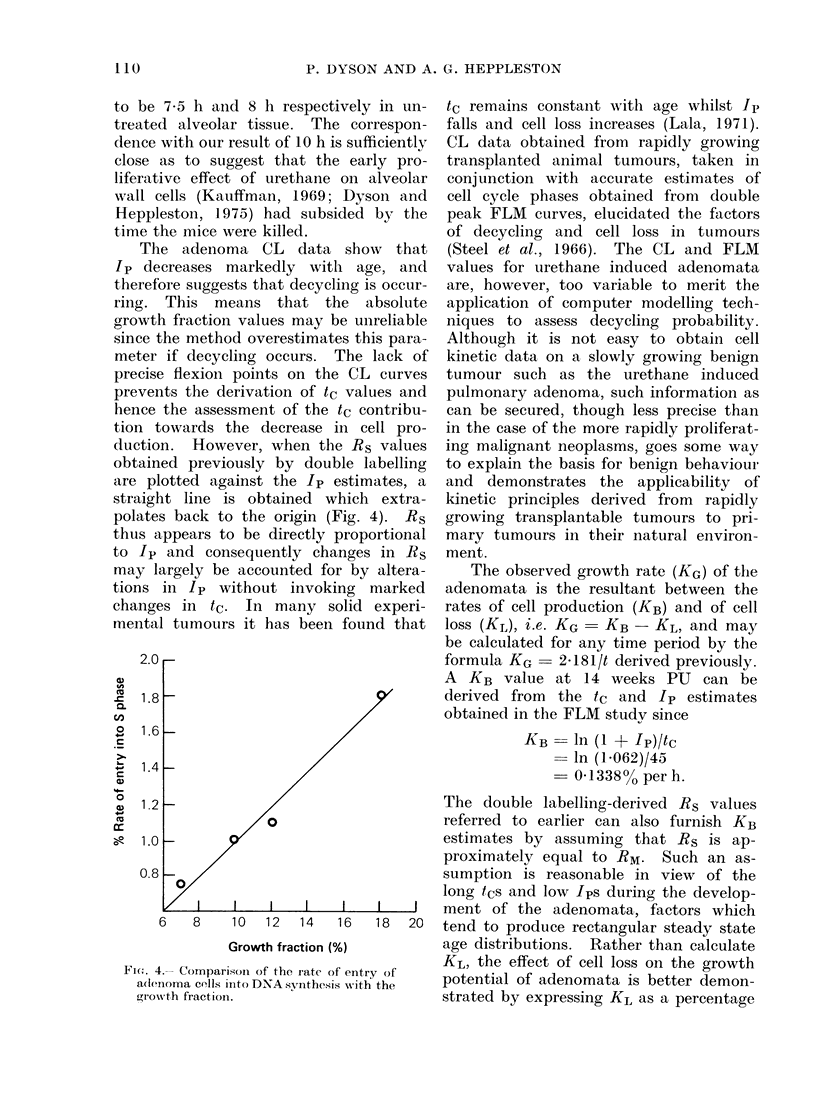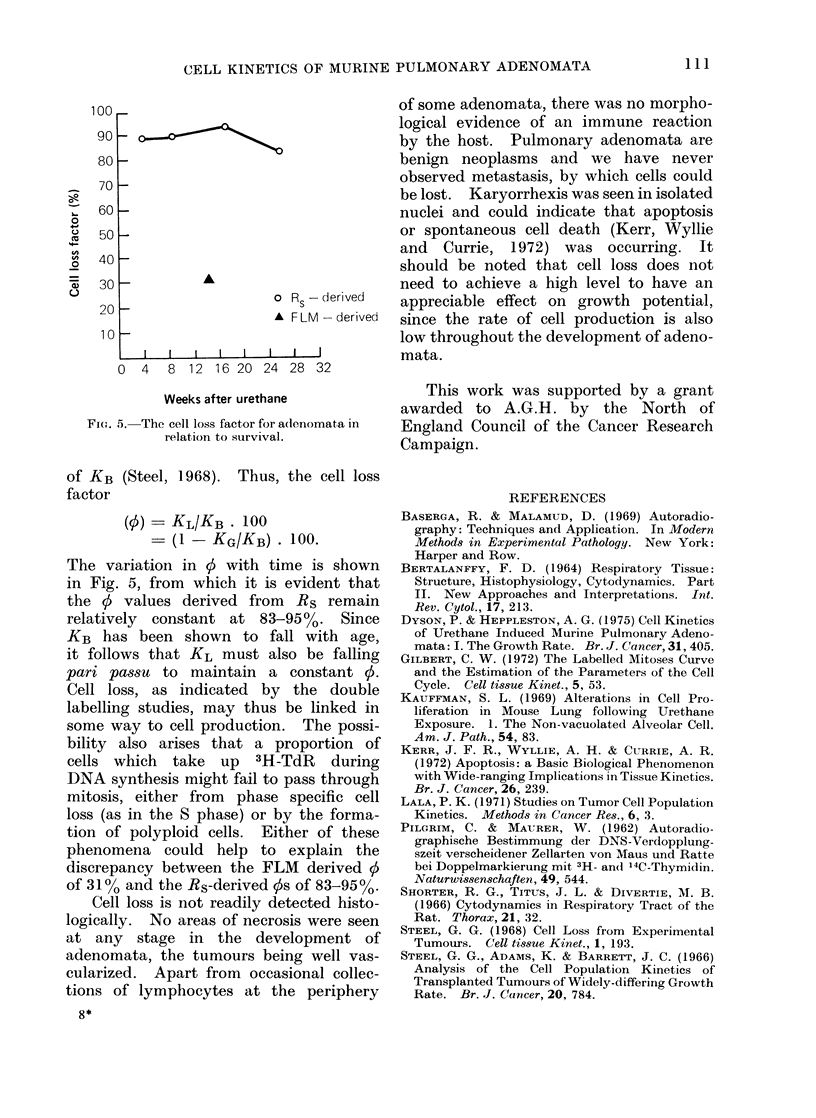# Cell kinetics of urethane-induced murine pulmonary adenomata: II, the growth fraction and cell loss factor.

**DOI:** 10.1038/bjc.1976.11

**Published:** 1976-01

**Authors:** P. Dyson, A. G. Heppleston

## Abstract

Continuous labelling of urethane induced pulmonary adenomata in adult male A2G mice at intervals up to 20 weeks showed that the growth fraction fell progressively from 18% at 7 weeks to 7% at 20 weeks. This fall appears to be wholly responsible for the decrease in production of adenoma cells with age. A fraction labelled mitoses curve was constructed for pulmonary adenomata at 14 weeks post urethane. Only the first peak was apparent, giving median t2 and ts values of 2 and 9 h respectively. The cell cycle time was calculated at 45 h and the growth fraction at 6-2%, whilst the cell loss factor was estimated at 31%. Other cell loss values were calculated from data on the rate of entry into DNA synthesis obtained previously by double labelling. These values remained constant with time at 83-95%, suggesting that cell loss is in some way linked to cell production. However, the development of polyploidy in adenoma cells could not be eliminated. No areas of necrosis were seen in the adenomata at any time although karyorrhexis occurred in isolated cells. The labelling characteristics of alveolar wall cells in the same lung sections as the adenomata did not vary with time and the continuous labelling curve gave a growth fraction of 1-8%, a DNA synthetic time of 10 h and a cell cycle time of 30 h.


*Br. J. Cancer* (1976) **33**, 105

### CELL KINETICS OF URETHANE-INDUCED MURINE PULMONARY ADENOMATA: II. THE GROWTH FRACTION AND CELL LOSS FACTOR

P. DYSON AND A. G. HEPPLESTON

*From the Department of Pathology, University of Newcastle upon Tyne*

Received 15 August 1975 Accepted 19 September 1975

**Summary.—Continuous labelling of urethane induced pulmonary adenomata in adult male A2G mice at intervals up to 20 weeks showed that the growth fraction fell progressively from 18% at 7 weeks to 7% at 20 weeks. This fall appears to be wholly responsible for the decrease in production of adenoma cells with age.**

**A fraction labelled mitoses curve was constructed for pulmonary adenomata at 14 weeks post urethane. Only the first peak was apparent, giving median $t_2$ and $t_S$ values of 2 and 9 h respectively. The cell cycle time was calculated at 45 h and the growth fraction at 6·2%, whilst the cell loss factor was estimated at 31%. Other cell loss values were calculated from data on the rate of entry into DNA synthesis obtained previously by double labelling. These values remained constant with time at 83–95%, suggesting that cell loss is in some way linked to cell production. However, the development of polyploidy in adenoma cells could not be eliminated. No areas of necrosis were seen in the adenomata at any time although karyorrhexis occurred in isolated cells.**

**The labelling characteristics of alveolar wall cells in the same lung sections as the adenomata did not vary with time and the continuous labelling curve gave a growth fraction of 1·8%, a DNA synthetic time of 10 h and a cell cycle time of 30 h.**

IN THE CELLS comprising urethane induced pulmonary adenomata the rates of entry into metaphase ($R_M$) and into DNA synthesis ($R_S$) decreased as survival of the mice lengthened (Dyson and Heppleston, 1975). This fall in adenoma cell production took place concurrently with a hyperbolic decrease in growth rate and a linear increase in the doubling time. On the available evidence, it was not possible to say whether cell production or cell loss was the more important factor influencing the growth of the neoplasms, or whether the fall in cell production was due primarily to a decrease in growth fraction ($I_P$), increase in cell cycle time ($t_C$) or both. The duration of DNA synthesis ($t_S$) increased in the adenomata as the mice aged, raising the possibility that $t_C$ might also lengthen. In an attempt to determine the effects of $t_C$ and $I_P$ on cell production, a series of continuous labelling experiments was performed at intervals during the development of the adenomata.

Continuous labelling (CL) experiments *in vivo* usually entail injection of tritiated thymidine ($^3$H-TdR) at regular intervals shorter than $t_S$ over a period greater than the combined durations of the $G2$, $M$ and $G1$ phases, *i.e.* $> t_1 + t_2$ (where $t_1 = G1 + 0{\cdot}5\ M$ and $t_2 = G2 + 0{\cdot}5\ M$). All cells entering DNA synthesis during this period become labelled and under ideal conditions there is a marked flexion point in the CL curve when all cycling cells are labelled. The time taken for $I_L$ to reach this flexion point is equal to $t_1 + t_2$; $t_S$ and $t_C$ may then be calculated using the simultaneous equations method (Baserga and Malamud, 1969). At the flexion point, the CL index gives an approximate estimate of $I_P$. If, however, significant decycling of labelled cells occurs $I_P$ will be over-estimated since non-proliferative labelled cells will be included; the lower $I_P$, the more decycling and the greater will be the overestimate. Taken in conjunction with information on the duration of cell cycle phases obtained from a fraction labelled mitoses (FLM) study, this $I_P$ value can be used in an iterative procedure to estimate decycling probabilities and so estimate $I_P$ more accurately (Steel, Adams and Barrett, 1966). The combining of FLM and CL data can also be used to test various theoretical models of cell loss.

The most direct and informative procedure for kinetic analysis of a cell population is by construction of a fraction labelled mitoses (FLM) curve. Ideally, this method requires a relatively high proliferative rate so that sufficient mitoses (usually about 100) can be counted at each sampling interval to give a precise curve. In the case of urethane induced adenomata the proliferative rate is high only during the first few weeks post urethane (PU), during which the neoplasms are small and difficult to find in lung sections. When the adenomata are large enough to be readily identified their proliferative rate is much reduced. As a compromise, it was considered that a FLM curve could most usefully be constructed at 14 weeks PU. The difference between the rate of adenoma cell production ($K_B$) and the observed growth rate ($K_G$) gives an estimate of the rate of cell loss ($K_L$) and an indication of its contribution to the diminishing growth rate.

### MATERIALS AND METHODS

Specific pathogen-free (SPF) male A2G mice (Laboratory Animals Centre, Carshalton) were injected intraperitoneally (i.p.) with urethane (British Drug Houses) at a dose of 1 mg/g body weight when 3–4 months old.

The CL experiments were performed at 7, 11, 14 and 20 weeks PU, using 10–11 mice for each experiment. $^3$H-TdR of specific activity 5 Ci/mmol (Radiochemical Centre, Amersham) was injected i.p. into each mouse (0·5 $\mu$Ci/g body weight) at 10.30 hrs and one animal killed 40 min later. The remaining mice received another $^3$H-TdR injection 5 h after the first, and again one mouse was killed 40 min later. This procedure was repeated at 5-hourly intervals until a total of 50 h of continuous labelling had been achieved in each experiment.

For the FLM experiment 42 male, SPF derived A2G mice survived 14 weeks after urethane treatment. They were then given a single i.p. injection of $^3$H-TdR at 10.30 hrs and killed at intervals over the succeeding 60 h.

The mice were killed by cervical dislocation, the left lung fixed in Carnoy's fluid and serial paraffin sections prepared for determination of the CL indices by stripping film autoradiography as described previously. Dipping film autoradiography with Ilford K2 emulsion was employed for the FLM study and at least 100 metaphases were counted at each sampling interval, an objective which often necessitated scanning several adenomata. The pulse labelling index ($I_S$) was determined by counting 3000 adenoma nuclei in mice killed 1–2 h after $^3$H-TdR injection.

### RESULTS

### *Continuous labelling*

The findings in the 7, 11, 14 and 20 week observations on *adenomata* are given in Fig. 1a, c, b and d respectively, the curves being drawn by eye. The lack of marked flexion points on the CL curves make it difficult to derive an exact $t_1 + t_2$ value, but it was judged to remain approximately constant at 35–40 h. The $I_L$ value at the flexion points, which approximates to $I_P$, fell as the adenomata aged. The $I_P$ estimates at 7, 11, 14 and 20 weeks were 18, 12, 10 and 7% respectively. These figures indicate the overall pattern of change though they probably overestimate $I_P$ and underestimate its rate of fall in view of the fact that decycling of labelled cells should be increasing as $I_P$ is progressively falling with time.

The CL pattern for *alveolar wall cells,* in the same lung sections as the adenomata, was essentially similar at the various time intervals and all the results were therefore combined (Fig. 2). At $t_1 + t_2$ of 20 h and an $I_P$ of 1·8% were taken from the point where the curve starts to flatten. Beyond this point the CL curve still has a slight positive gradient, suggesting either that some decycling of labelled cells occurred or that labelled leucocytes were emigrating from capillaries into the alveolar wall. Assuming that the alveolar wall tissue was in steady state during the CL experiments, $t_S$ and $t_C$ may be calculated from 2 equations: $t_C = t_S + (t_1 + t_2)$, and the pulse labelling index $(I_S) = t_S \cdot I_P/t_C$. Taking the 40 min $I_L$ value of 0·6% as equivalent to $I_S$, $t_S$ can be calculated

FIG 1 (a)

FIG 1 (b)

FIG 1 (c)

FIG 1 (d)

FIG. 1.—Continuous labelling curves for adenomata at various intervals after urethane treatment: (a) 7 weeks, (b) 11 weeks, (c) 14 weeks and (d) 20 weeks.

at 10 h and $t_C$ at 30 h since $t_1 + t_2$ was estimated to be 20 h.

*Fraction labelled mitoses*

The *adenoma* curve (Fig. 3) was first drawn by eye but a similar curve was then obtained by means of a Gilbert (1972) computer programme. Only the first peak is apparent, the curve rapidly flattening from about 20 h, indicating that there is considerable variability in $t_1$ between adenoma cells, and that the initially synchronized cohort of labelled cells has rapidly become randomly distributed throughout the component phases of the cell cycle. Compared with the variability in $t_1$, the precision of the peak shows that there is little variation in $t_2$ and $t_S$. The FLM curve gives median $t_2$ and $t_S$ values of 2 and 9 h respectively. The plateau FLM value of 0·20 represents the ratio of $t_S$ to $t_C$, *i.e.* the theoretical labelling index ($I_{S(T)}$) for cycling cells alone. $t_C$ by calculation is therefore 45 h and $t$, by subtraction, 34 h. The observed pulse labelling index

FIG. 2.—Continuous labelling curve of alveolar wall cells from the combined results at all intervals.

FIG. 3.—Fraction labelled mitoses curve for adenomata 14 weeks after urethane injection.

($I_S$) was found to be 1·23% (s.e. $\pm$ 0·10%), and $I_P$ was then calculated at 6·2% from the ratio of $I_S$ to $I_{S(T)}$.

DISCUSSION

Although *alveolar walls* include more than one cell population undergoing renewal, it is believed that most of the mitotic activity (70–80%) is contributed by the non-vacuolated cells (Bertalanffy, 1964; Kauffman, 1969) which evidently correspond to alveolar macrophage precursors. Thus the $t_S$ of 10 h and the $t_C$ of 30 h are probably applicable to monocytes or promonocytes. Pilgrim and Maurer (1962) by double isotope labelling and Shorter, Titus and Divertie (1966) by a limited FLM study estimated $t_S$

to be 7·5 h and 8 h respectively in untreated alveolar tissue. The correspondence with our result of 10 h is sufficiently close as to suggest that the early proliferative effect of urethane on alveolar wall cells (Kauffman, 1969; Dyson and Heppleston, 1975) had subsided by the time the mice were killed.

The adenoma CL data show that $I_P$ decreases markedly with age, and therefore suggests that decycling is occurring. This means that the absolute growth fraction values may be unreliable since the method overestimates this parameter if decycling occurs. The lack of precise flexion points on the CL curves prevents the derivation of $t_C$ values and hence the assessment of the $t_C$ contribution towards the decrease in cell production. However, when the $R_S$ values obtained previously by double labelling are plotted against the $I_P$ estimates, a straight line is obtained which extrapolates back to the origin (Fig. 4). $R_S$ thus appears to be directly proportional to $I_P$ and consequently changes in $R_S$ may largely be accounted for by alterations in $I_P$ without invoking marked changes in $t_C$. In many solid experimental tumours it has been found that

FIG. 4.—Comparison of the rate of entry of adenoma cells into DNA synthesis with the growth fraction.

$t_C$ remains constant with age whilst $I_P$ falls and cell loss increases (Lala, 1971). CL data obtained from rapidly growing transplanted animal tumours, taken in conjunction with accurate estimates of cell cycle phases obtained from double peak FLM curves, elucidated the factors of decycling and cell loss in tumours (Steel *et al.*, 1966). The CL and FLM values for urethane induced adenomata are, however, too variable to merit the application of computer modelling techniques to assess decycling probability. Although it is not easy to obtain cell kinetic data on a slowly growing benign tumour such as the urethane induced pulmonary adenoma, such information as can be secured, though less precise than in the case of the more rapidly proliferating malignant neoplasms, goes some way to explain the basis for benign behaviour and demonstrates the applicability of kinetic principles derived from rapidly growing transplantable tumours to primary tumours in their natural environment.

The observed growth rate ($K_G$) of the adenomata is the resultant between the rates of cell production ($K_B$) and of cell loss ($K_L$), *i.e.* $K_G = K_B - K_L$, and may be calculated for any time period by the formula $K_G = 2{\cdot}181/t$ derived previously. A $K_B$ value at 14 weeks PU can be derived from the $t_C$ and $I_P$ estimates obtained in the FLM study since

$$\begin{aligned} K_B &= \ln\,(1 + I_P)/t_C \\ &= \ln\,(1{\cdot}062)/45 \\ &= 0{\cdot}1338\%\ \text{per h}. \end{aligned}$$

The double labelling-derived $R_S$ values referred to earlier can also furnish $K_B$ estimates by assuming that $R_S$ is approximately equal to $R_M$. Such an assumption is reasonable in view of the long $t_C$s and low $I_P$s during the development of the adenomata, factors which tend to produce rectangular steady state age distributions. Rather than calculate $K_L$, the effect of cell loss on the growth potential of adenomata is better demonstrated by expressing $K_L$ as a percentage

FIG. 5.—The cell loss factor for adenomata in relation to survival.

of $K_B$ (Steel, 1968). Thus, the cell loss factor

$$(\phi) = K_L/K_B \cdot 100$$
$$= (1 - K_G/K_B) \cdot 100.$$

The variation in $\phi$ with time is shown in Fig. 5, from which it is evident that the $\phi$ values derived from $R_S$ remain relatively constant at 83–95%. Since $K_B$ has been shown to fall with age, it follows that $K_L$ must also be falling *pari passu* to maintain a constant $\phi$. Cell loss, as indicated by the double labelling studies, may thus be linked in some way to cell production. The possibility also arises that a proportion of cells which take up $^3$H-TdR during DNA synthesis might fail to pass through mitosis, either from phase specific cell loss (as in the S phase) or by the formation of polyploid cells. Either of these phenomena could help to explain the discrepancy between the FLM derived $\phi$ of 31% and the $R_S$-derived $\phi$s of 83–95%.

Cell loss is not readily detected histologically. No areas of necrosis were seen at any stage in the development of adenomata, the tumours being well vascularized. Apart from occasional collections of lymphocytes at the periphery of some adenomata, there was no morphological evidence of an immune reaction by the host. Pulmonary adenomata are benign neoplasms and we have never observed metastasis, by which cells could be lost. Karyorrhexis was seen in isolated nuclei and could indicate that apoptosis or spontaneous cell death (Kerr, Wyllie and Currie, 1972) was occurring. It should be noted that cell loss does not need to achieve a high level to have an appreciable effect on growth potential, since the rate of cell production is also low throughout the development of adenomata.

This work was supported by a grant awarded to A.G.H. by the North of England Council of the Cancer Research Campaign.

REFERENCES

BASERGA, R. & MALAMUD, D. (1969) Autoradiography: Techniques and Application. In *Modern Methods in Experimental Pathology*. New York: Harper and Row.

BERTALANFFY, F. D. (1964) Respiratory Tissue: Structure, Histophysiology, Cytodynamics. Part II. New Approaches and Interpretations. *Int. Rev. Cytol.*, **17**, 213.

DYSON, P. & HEPPLESTON, A. G. (1975) Cell Kinetics of Urethane Induced Murine Pulmonary Adenomata: I. The Growth Rate. *Br. J. Cancer*, **31**, 405.

GILBERT, C. W. (1972) The Labelled Mitoses Curve and the Estimation of the Parameters of the Cell Cycle. *Cell tissue Kinet.*, **5**, 53.

KAUFFMAN, S. L. (1969) Alterations in Cell Proliferation in Mouse Lung following Urethane Exposure. 1. The Non-vacuolated Alveolar Cell. *Am. J. Path.*, **54**, 83.

KERR, J. F. R., WYLLIE, A. H. & CURRIE, A. R. (1972) Apoptosis: a Basic Biological Phenomenon with Wide-ranging Implications in Tissue Kinetics. *Br. J. Cancer*, **26**, 239.

LALA, P. K. (1971) Studies on Tumor Cell Population Kinetics. *Methods in Cancer Res.*, **6**, 3.

PILGRIM, C. & MAURER, W. (1962) Autoradiographische Bestimmung der DNS-Verdopplungszeit verscheidener Zellarten von Maus und Ratte bei Doppelmarkierung mit $^3$H- and $^{14}$C-Thymidin. *Naturwissenschaften*, **49**, 544.

SHORTER, R. G., TITUS, J. L. & DIVERTIE, M. B. (1966) Cytodynamics in Respiratory Tract of the Rat. *Thorax*, **21**, 32.

STEEL, G. G. (1968) Cell Loss from Experimental Tumours. *Cell tissue Kinet.*, **1**, 193.

STEEL, G. G., ADAMS, K. & BARRETT, J. C. (1966) Analysis of the Cell Population Kinetics of Transplanted Tumours of Widely-differing Growth Rate. *Br. J. Cancer*, **20**, 784.